# Treatment Parameters Optimization to Compensate for Interfractional Anatomy Variability and Intrafractional Tumor Motion

**DOI:** 10.3389/fonc.2015.00291

**Published:** 2015-12-24

**Authors:** Romain Brevet, Daniel Richter, Christian Graeff, Marco Durante, Christoph Bert

**Affiliations:** ^1^GSI Helmholtzzentrum für Schwerionenforschung, Darmstadt, Germany; ^2^FAU Erlangen-Nürnberg and Universitätsklinikum Erlangen, Erlangen, Germany

**Keywords:** medical physics, radiotherapy, particle therapy, ions, treatment planning, moving targets, moving organs

## Abstract

Scanned ion beam therapy of lung tumors is severely limited in its clinical applicability by intrafractional organ motion, interference effects between beam and tumor motion (interplay), as well as interfractional anatomic changes. To compensate for dose deterioration caused by intrafractional motion, motion mitigation techniques, such as gating, have been developed. However, optimization of the treatment parameters is needed to further improve target dose coverage and normal tissue sparing. The aim of this study was to determine treatment-planning parameters that permit to recover good target coverage for each fraction of lung tumor treatments. For 9 lung tumor patients from MD Anderson Cancer Center (Houston, Texas), a total of 70 weekly time-resolved computed tomography (4DCT) datasets, which depict the evolution of the patient anatomy over the several fractions of the treatment, were available. Using the GSI in-house treatment planning system TRiP4D, 4D simulations were performed on each weekly 4DCT for each patient using gating and optimization of a single treatment plan based on a planning CT acquired prior to treatment. The impact on target dose coverage (*V*_95%,CTV_) of variations in focus size and length of the gating window, as well as different additional margins and the number of fields was analyzed. It appeared that interfractional variability could potentially have a larger impact on *V*_95%,CTV_ than intrafractional motion. However, among the investigated parameters, the use of a large beam spot size, a short gating window, additional margins, and multiple fields permitted to obtain an average *V*_95%,CTV_ of 96.5%. In the presented study, it was shown that optimized treatment parameters have an important impact on target dose coverage in the treatment of moving tumors. Indeed, intrafractional motion occurring during the treatment of lung tumors and interfractional variability were best mitigated using a large focus, a short gating window, additional margins, and three fields.

## 1 Introduction

Treating moving targets, such as non-small cell lung cancer (NSCLC) tumors, using photon radiation therapy has been investigated (1) and is being clinically used nowadays combined to real-time tracking (2, 3). However, using heavy-ion scanned beam therapy has shown many advantages compared to conventional radiotherapy (4, 5) by reducing the number of fields, which have to be used as well as the dose delivered to the organs at risk (OARs) in the vicinity of the tumor. It also demands high precision and accuracy when applied to moving tumors because of the possible dose delivery errors induced by range shifts themselves due to intrafractional motion, interfractional anatomic changes, and patient misalignments (6, 7). This is why several motion mitigation techniques, such as gating, rescanning, or tracking have been developed and are still under development (8). Gating (9, 10) is a technique which consists in turning the beam on when the moving tumor reaches a precise motion state, in general, at the end of exhalation while the tumor is the most stable. It has shown great potential and has thus been successfully used in Japan in ion beam therapy with passive absorbers for beam shaping (11–13). Active scanned beam delivery introduces interplay effects (14) and even though tumor motion mitigation techniques are used, these effects can lead to non-conformal dose delivery. In order to address specifically this problem, 4D treatment planning systems (4DTPS) have been implemented (15, 16) and permit to simulate the treatment of moving targets using gating while also taking interplay effects into account. Nonetheless, treatment parameters still have to be optimized to maximize motion mitigation obtained using gating. Several studies have been performed to determine the influence of different parameters on the dose delivery: Bert et al. (17) proposed to increase pencil beam overlap to mitigate interplay effects as well as Steidl (18) and Richter (19) whose studies displayed the effects of different lateral grid spacing, isoenergy slice distance, focus size, and Bragg peak width. In a combination gating and rescanning, Furukawa et al. (20) proposed a method called phase-controlled rescanning, aiming at compensating further the residual tumor motion within the gating window. Rescanning was used as mitigation technique by Knopf et al. (21), and the impact of the entry channel was also investigated through different field scenarios. Target definition including tumor motion, size, and position (22), as well as range-adapted margins, were discussed (15, 23–25) and implemented (26). However, those studies concentrated on intrafractional motion compensation, meaning that the possible anatomic variability between the time of the treatment planning CT and treatment or also between fractions was not taken into account. Simulations were, in general, restricted to a single 4DCT taken for treatment planning. The purpose of this study was to investigate which parameters could be isolated and optimized in order to compensate correctly for both intrafractional tumor motion and interfractional anatomic changes and/or patient misalignments. To this end, in a cohort of patients with a time series of 4DCTs and for different combinations of treatment and/or beam parameters, one gating plan was optimized using the first weekly 4DCT of each patient and was forward calculated on the successive 4DCTs of the weeks following treatment planning. Results were then compared to determine the best configuration.

## 2 Materials and Methods

### 2.1 Patient Cohort

Data from 9 NSCLC lung tumor patients from the MD Anderson Cancer Center (MDACC) (27) were used to perform this study, reaching a total of 70 weekly 4DCT datasets. Each 4DCT was composed of 10 3DCTs representing 10 different phase-based tumor motion phases over the breathing cycle. End-exhale, referred to as phase n° 5, was set as the reference state. Number of weeks, motion amplitude, angles for single field and multiple fields calculations, and clinical target volumes (CTVs) with and without additional margins are listed in Table 1. The number of weekly 4DCTs per patient varied between 6 and 10; each 4DCT was treated as a single fraction, with the first 4DCT as the planning CT. Most of the patients have an average tumor motion below 5 mm and only one patient shows a tumor motion above 20 mm (Patient 9).

**Table 1:** **Description of the 9 NSCLC patients from MDACC (see Figure 1 for field angles illustration): patient number, number of weeks available, mean motion amplitude and range, field angle for single field calculations (SFUD), field angles for multiple fields calculations (SFUD1, 2, and 3), volume of the CTV, and volumes of the extended target: 3 mm isotropic (I3), 3 mm + 3% range (R3), and combination of both (I3 + R3)**.

Patient	Weeks	Motion (mm)	Angles (°)				Volumes (cc)			
			SFUD	SFUD1	SFUD2	SFUD3	CTV	I3	R3	I3 + R3
1	8	3.4	240	180	225	270	236	322	406	518
2	6	8.6	0	0	315	270	574	718	891	1057
3	9	10.1	0	270	315	0	161	213	335	409
4	8	3.3	225	180	225	270	676	819	925	1089
5	10	4.1	0	0	315	270	372	472	648	791
6	8	1.8	0	0	315	270	705	828	935	1072
7	7	1.6	180	180	225	270	124	172	253	322
8	8	4	180	180	225	270	45	65	102	133
9	6	23.5	180	180	225	270	125	164	203	250

### 2.2 Treatment Planning

#### 2.2.1 Image Registration

Rigid registration of reference phases of each subsequent CT was performed to mimic patient setup and alignment. Then non-rigid registration was used between each 4DCT motion phase using Plastimatch (28). For each patient, clinical target volumes (CTVs) as well as OAR contours (esophagus, heart, and spinal cord) were provided by physicians of MDACC for the first weekly 4DCT. Lung contours were extracted from the weekly 4DCTs using an in-house algorithm. Files containing vector fields (between the first week and the following ones) obtained using deformable registration (29) were then used to propagate the previously mentioned contours from the reference phase of the first weekly CT to the reference phases of the following ones (16). Finally, vector field files yielded by deformable registration applied on the 10 states of each weekly 4DCT permitted to propagate the contours from the reference state to the 9 other motion states.

#### 2.2.2 Optimization and 4D Calculations

In this study, the technique used to mitigate motion was gating. All gating plans were simulated using ^12^C ions and the GSI treatment planning system TRiP4D (16), based on TRiP98 and modified to allow 4D-dose calculations. For each patient, plans were initially optimized to the internal target volume (ITV) of the first week’s CT using one unique planned dose of 8.1 Gy(RBE). Motion-related geometrical and range changes were considered according to Graeff et al. (26). The generated raster scanning plan was then used for all 4D calculations of the first week itself and the following ones as well. It means that only one plan was used per patient and that there was no replanning before simulations of the fractions following the first optimized one. In each case, the ITV was built using a combination of five CTVs (26) from five different motion phases representing 25% of the amplitude. The motion surrogate was defined according to Lujan et al. (30), i.e., a sine to the power of 4 with a unique period of 3.6 s. Only one starting phase (0°) was studied because, due to gating, beam delivery for different starting phases is quickly synchronized after the first few spills of the synchrotron accelerator, thus calculations yield very similar results for different starting phases. As other fixed treatment parameters, the distance between each raster position was set to 2 mm on each isoenergy slice (IES), and the distance between two IESs was set to 3 mm water equivalent using a ripple filter of 3 mm (31).

#### 2.2.3 Investigated Parameters

**Figure 1:**
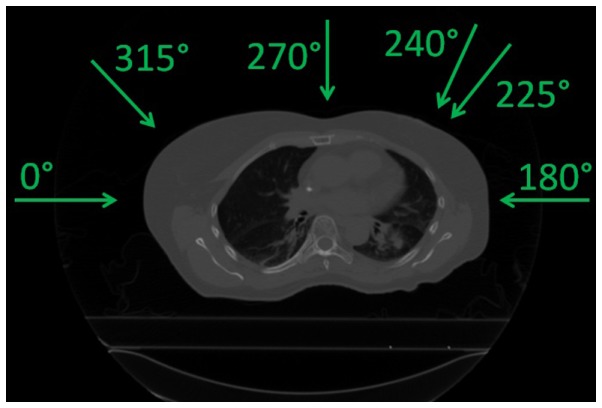
**Field angles used (see Table 1)**.

The impact of different treatment plan parameters on the dose delivery was investigated using the field angles listed in Table 1. First, using one single field (see column “SFUD” of Table 1 and Figure 1) and ITV margins only, variations in focus size and length of the gating window (GW) were performed. Three GWs: 11.9, 30, and 50% of the amplitude and three beam foci: 6, 10, and 15 mm (FWHM) were chosen as varying parameters. Two configurations in particular were compared:

- *LFSG*: large focus (15 mm) and short GW (11.9%),
- *SFLG*: small focus (6 mm) and long GW (50%).

**Figure 2:**
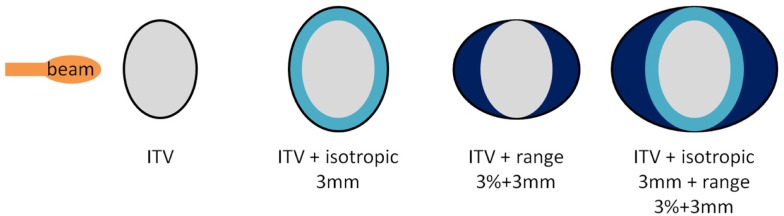
**Different margins cases used in the second part of the study**. From the left to the right, configurations displayed are referred to as ITV, I3, R3, and I3 + R3.

As a second part, using the same single field angles, different planning target volumes (PTV) created by adding additional margins to the originally optimized plans were also investigated as another solution to recover good target coverage. Three different cases were studied: 3 mm isotropic margins (geometrical, referred to as I3), 3 mm + 3% range margins (water equivalent, referred to as R3), and combination of both (referred to as I3 + R3, see Figure 2). Resulting dose deliveries were compared to the results obtained using ITV margins only. Combinations of GWs and foci (same 3 foci and 3 GWs than in the previous paragraph) were again investigated in each case to observe the impact of additional margins on the range. Finally, still using the 9 possible GW/focus combinations, the number of fields was varied from 1 to 3 (see Table 1 for field angle values, columns “SFUD1” to “SFUD3” and Figure 1) using only ITV margins first and then using the additional PTV margins which had been determined to yield the best results in the second section of this chapter, resulting in the following cases:

- *SFITV*: single field to ITV only,
- *SFPTV*: single field to the isotropic/range margins (same as I3 + R3),
- *2FITV*: ITV only but with 2 fields,
- *2FPTV*: same margins as SFPTV but with 2 fields,
- *3FITV*: ITV only but with 3 fields,
- *3FPTV*: same margins as SFPTV but with 3 fields.

### 2.3 Data Analysis

In each case, the dose distribution of each week was obtained by accumulating the dose delivered to each motion state on the reference phase of the 4DCTs using state-to-state non-rigid vector fields. To estimate the impact of each previously described parameter and configuration on the dose delivery, the following two indexes were used:

- *Target coverage*, *V*_95_: volume of the target to which 95% of the planned dose is delivered, representing the quality of target dose coverage, unit is percentage of volume,
- *Conformity number (CN)* (32): allowing a quantification of the high-dose regions inside and outside the tumor (the higher, the better) and defined by:
  (1) $$\mathrm{CN}=\frac{V_{95\%,\text{CTV}}}{V_{\text{CTV}}}\times \frac{V_{95\%,\text{CTV}}}{V_{95\%}}$$
  where *V*_95%,CTV_ is the *V*_95_ value defined above, *V*_CTV_ the volume of the CTV, and *V*_95%_ the total volume which receives at least 95% of the dose.

The main focus of this study is the impact of treatment plan parameters on dose delivery. All dose calculations were computed for weekly simulations but not for the cumulated total treatment regime. Therefore, OAR limit dose values from the literature were not taken into account but are used only a general indicator of plan quality. This study aims at determining clearly the effect of the investigated parameters on the decreased quality of the dose delivery due to both interfractional anatomy changes and intrafractional tumor motion. In each case, a Wilcoxon signed-rank test was performed using a level of significance of 0.05 to estimate the difference between two sets of datapoints. In the case of samples containing more than 10 values, the *p*-value (*p*) was computed using the obtained z-score (*z*).

## 3 Results

All simulations were performed on the weekly 4DCTs with a planned dose of 8.1 Gy(RBE), which corresponds to a single field dose as reported by NIRS (5) according to LEM IV (33). In all the following figures, the average value (marker), the median value (horizontal bar in the box), the 25th and 75th percentile, and the total range of all values are given. In some cases, different types of simulations were studied and referred to as

- *3D0 simulations:* planned, static dose simulations using the first weekly CT (week 0 in reference phase),
- *4D0 simulations:* 4D dose simulations using the first weekly 4DCT (week 0) and the same plan than 3D0 simulations, which contains the effects of intrafractional motion only,
- *4DN simulations:* 4D dose simulations using all the following weekly 4DCTs (weeks 1 to 5–9) and the same plan than 3D0 simulations, which contains the effects of both intrafractional motion and interfractional patient anatomic changes.

### 3.1 Beam Focus and Gating Window

**Figure 3:**
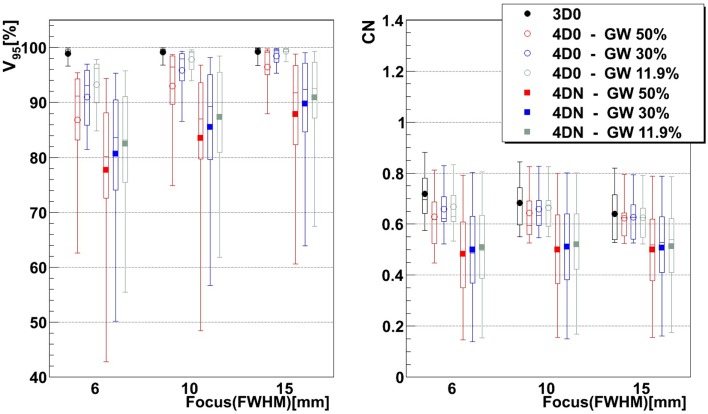
**Impact of the focus and the GW on *V*_95_ and CN**. Each 3D0 and 4D0 bar is composed of 9 points and each 4DN bar of 61 points.

**Figure 4:**
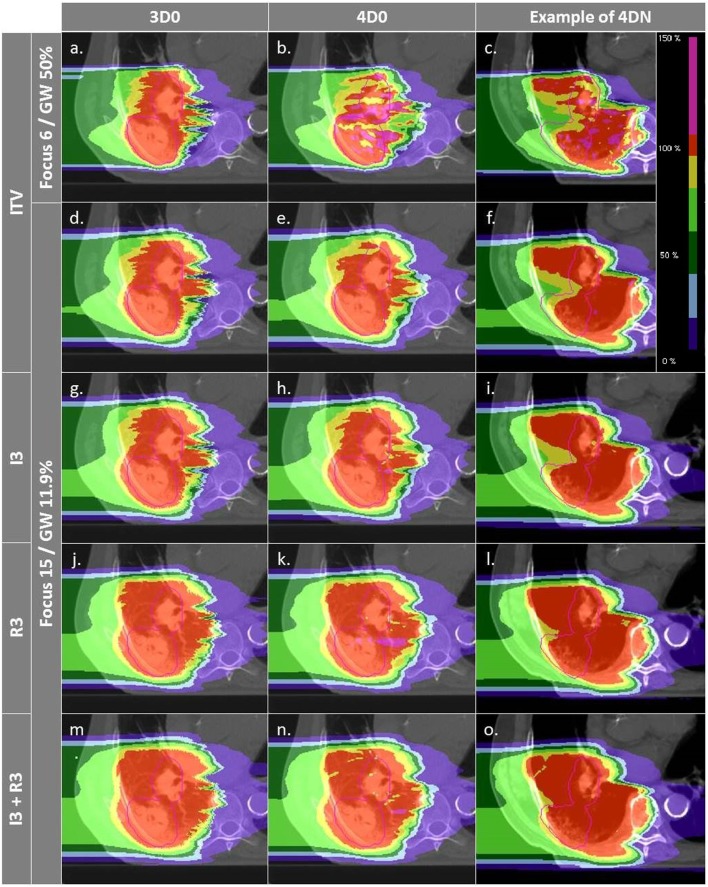
**Dose distributions of patient 3 using different weekly CTs**. Pink contours represent the CTV (week 6). Cases **(A–C)** and **(D–F)** are obtained using the SFLG and the LFSG configurations, respectively, simulations (week 6). Cases **(A–F)** are obtained using the SFLG and the LFSG configurations, respectively, with ITV margins only. Examples **(G–P)** are obtained using the LFSG configuration and 3 mm isotropic margins, 3 mm + 3% range margins, and the combination of both previous margins, respectively.

Figure 3 and Table 2 show *V*_95_ and CN for different GW/focus combinations, for all patients. 3D simulations show good results for all focus sizes, with slightly better target coverage and slightly worse conformity for the largest focus (*p* < 0.05). The 4D0 simulations show the effect of intrafractional tumor motion on target dose coverage: *V*_95_ decreases with a large variability for the smaller focus sizes. A large focus and gating window of 30% restores target coverage to the static values. CN, however, shows no significant (*p* < 0.05) change with GW or focus but decreases slightly compared to static calculations. 4DN results of the following weeks permit to investigate the effect of both interfractional changes but also intrafractional motion. Comparison to 4D0 shows a similar trend for GW and focus size, but the interfractional changes result in approximately 10 worse target coverage and CN. Without margins, adequate target coverage cannot be reached for any simulation with a small focus/large GW and less for than half with a large focus/small GW. Dose cuts for these combinations are displayed for the 7th weekly CT of patient 3 in Figures 4A–F, respectively.

**Table 2:** **Results of the influence of the focus and gating window on *V*_95_ and CN**.

Simulations	Focus (mm)	Gating window (%)	*V*_95_ (%)			CN			*V*_95_ > 95% (%)
			Min.	Mean	Max.	Min.	Mean	Max.	
3D0	6	–	96.6	98.9	99.9	0.57	0.72	0.88	100
	10	–	96.8	99.1	99.9	0.55	0.68	0.84	100
	15	–	96.7	99.2	99.9	0.53	0.64	0.82	100
4D0	6	50	62.6	86.8	95.4	0.45	0.63	0.81	11.1
		30	81.4	91.0	97.0	0.52	0.66	0.83	33.3
		11.9	84.9	93.2	97.9	0.53	0.67	0.83	55.6
	10	50	74.8	93.0	98.7	0.53	0.64	0.83	66.7
		30	86.5	95.9	99.3	0.55	0.66	0.83	66.7
		11.9	94.0	97.9	99.6	0.55	0.66	0.83	88.9
	15	50	88.0	96.5	99.8	0.52	0.62	0.80	77.8
		30	95.4	98.5	99.8	0.53	0.63	0.79	100
		11.9	97.4	99.3	99.9	0.52	0.63	0.79	100
4DN	6	50	42.8	77.7	94.3	0.15	0.48	0.79	0
		30	50.2	80.6	95.4	0.14	0.50	0.80	1.6
		11.9	55.4	82.5	95.8	0.15	0.51	0.81	4.9
	10	50	48.4	83.5	96.8	0.16	0.50	0.80	11.5
		30	56.7	85.5	98.2	0.15	0.51	0.80	27.9
		11.9	61.8	87.4	98.5	0.17	0.52	0.80	29.5
	15	50	60.6	87.9	98.8	0.16	0.50	0.79	37.7
		30	63.9	89.8	99.1	0.16	0.51	0.79	41
		11.9	67.5	90.9	99.3	0.17	0.51	0.79	42.6

### 3.2 Margins

**Figure 5:**
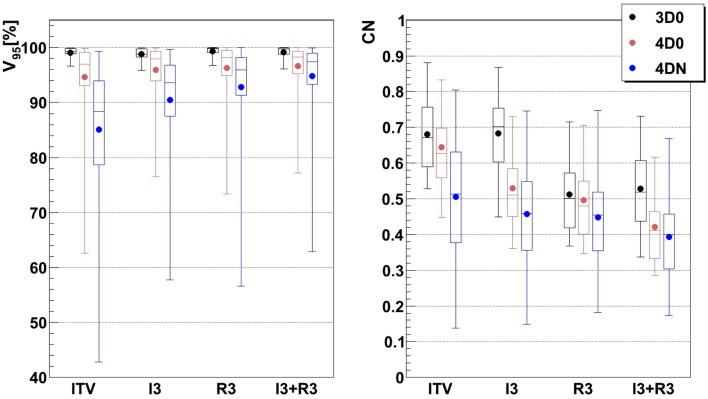
**Impact of additional margins on *V*_95_ and CN: ITV (ITV margins only), I3 (3 mm isotropic margins), R3 (3 mm + 3% range margins), and I3 + R3 (3 mm isotropic + 3 mm + 3% range margins)**. Each 3D0 bar is composed of results obtained using all 9 patients and 3 foci, representing 27 points, and each 4D0 and 4DN bar are composed of results obtained using all 9 patients, 3 foci, and 3 GWs, representing 81 and 549 points, respectively.

Not surprisingly, margins are necessary to achieve target coverage including interfractional changes. Figure 5 and Table 3 show the impact on *V*_95_ and CN for ITV only (ITV), ITV + 3 mm isotropic margins (I3), ITV + 3% + 3 mm range margins (R3), and a combination of both margins (I3 + R3), respectively. 3D0 simulations reveal that range margins have a larger impact on CN than isotropic ones (*p* < 0.05), as shown in Table 3. Calculations on the planning CT (4D0) show a minor but significant impact from increased margins. CN is degraded through increasing margins (*p* < 0.05). Interestingly, the isotropic margins show a comparable effect to range margins in the 4D calculations, as opposed to 3D. As a consequence, also the combined margins further decrease CN. The margin size shows a considerable impact on interfractional changes, decreasing the target coverage gap between 4D0 and 4DN from 10 to 2% with the I3 + R3 combination. Range margins are more effective than isotropic margins (*p* < 0.05). The same trend can be observed for CN, which decreases with margin size (*p* < 0.05), but reaches nearly the level of 4D0 for I3 + R3. The percentage of successful fractions (*V*_95_ > 95%) reaches 68.7% on average for I3 + R3, but 90.2% for the I3 + R3 for the large focus/small GW. Exemplary dose distributions (using the 1st and again the 7th weekly CTs of patient 3) using the largest focus and the shortest GW combined to ITV margins (ITV), and to ITV plus I3, R3, and I3 + R3 are displayed in Figures 4D–O, respectively.

**Table 3:** **Results of the influence of ITV margins (ITV), 3 mm isotropic margins (I3), 3 mm + 3% range margins (R3), and a combination of the two last ones (I3 + R3) on *V*_95_ and CN**.

Simulations	Margins	*V*_95_ (%)			CN			*V*_95_ > 95% (%)
		Min.	Mean	Max.	Min.	Mean	Max.	
3D0	ITV	96.6	99.1	99.9	0.53	0.68	0.88	100
	I3	95.9	98.8	100	0.45	0.68	0.87	100
	R3	96.7	99.4	100	0.37	0.51	0.72	100
	I3 + R3	96.2	99.1	100	0.34	0.53	0.73	100
4D0	ITV	62.6	94.7	99.9	0.45	0.64	0.83	66.7
	I3	76.5	95.9	100	0.36	0.53	0.73	70.4
	R3	73.4	96.3	100	0.35	0.50	0.70	74.1
	I3 + R3	77.2	96.6	100	0.29	0.42	0.62	75.3
4DN	ITV	42.8	85.1	99.3	0.14	0.50	0.81	21.9
	I3	57.8	90.5	99.7	0.15	0.46	0.75	40.4
	R3	56.6	92.8	100	0.18	0.45	0.75	56.3
	I3 + R3	62.8	94.8	100	0.17	0.39	0.67	68.7

*Each margins case of the 3D0 calculations was done using the 3 foci described previously. In the case of 4D0 and 4DN values, the 9 possible focus/GW combinations presented previously were used*.

### 3.3 Number of Fields

**Figure 6:**
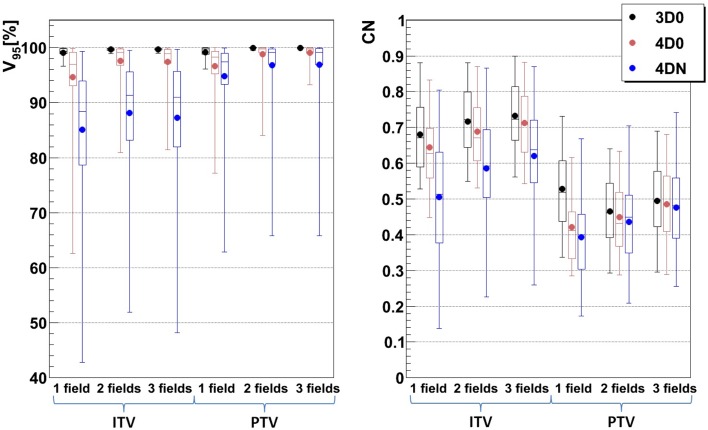
**Impact of different numbers of fields on *V*_95_ and CN: 1, 2, and 3 fields with ITV (ITV margins only) and PTV (3 mm isotropic + 3 mm + 3% range margins)**. Each 3D0 bar is composed of results obtained using all 9 patients and 3 foci, representing 27 points, and each 4D0 and 4DN bar are composed of results obtained using all 9 patients, 3 foci, and 3 GWs, representing 81 and 549 points, respectively.

**Figure 7:**
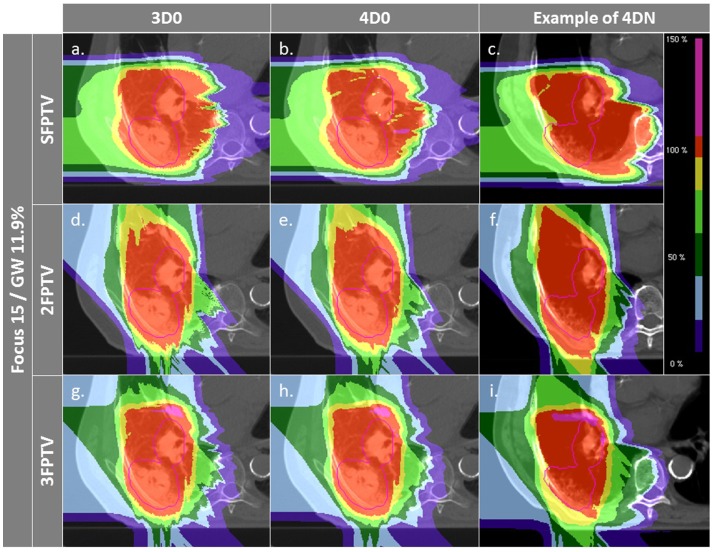
**Dose distributions of patient 3 using different weekly CTs**. Pink contours represent the CTV. First column displays 3D0 simulations, second column 4D0 simulations, and the third one 4DN simulations using week 6. Cases **(A–I)** are obtained using the LFSG configuration combined to isotropic and range margins and one, two, and three fields, respectively.

Figure 6 and Table 4 show the impact of the number of fields for the ITV only and for ITV with I3 + R3, subsequently called PTV. Again, 3D0 simulations yield excellent target coverage, but multiple fields slightly improve CN for the ITV only, while they degrade CN for PTV margins (*p* < 0.05, see Table 4). Using more than one field helps to mitigate intrafractional motion, with increasing *V*_95_ for both ITV and PTV margins (*p* < 0.05). PTV margins improve target coverage but considerably decrease CN (both *p* < 0.05). The same effect can be observed for 4DN, where more than one field and PTV margins significantly increase *V*_95_. The conformity can essentially be restored to the static or 4D0 value using PTV margins and three fields. For this combination, more than 80% of simulations lead to adequate target coverage, and 93.4% for the small focus/large GW, see also Figure 6. A dose distribution example using week 6 of patient 3 from single field ITV simulations (SFITV) and single field, 2 fields, and 3 fields PTV simulations (SFPTV, 2FPTV, and 3FPTV) are shown in Figures 4D–F and 7A–F, respectively.

**Table 4:** **Results of the influence of ITV and PTV margins and of different numbers of fields on *V*_95_ and CN**.

Simulations	Margins	Fields	*V*_95_ (%)			CN			*V*_95_ > 95% (%)
			Min.	Mean	Max.	Min.	Mean	Max.	
3D0	ITV	1	96.6	99.1	99.9	0.53	0.68	0.88	100
		2	98.9	99.7	100	0.55	0.72	0.88	100
		3	99.0	99.7	100	0.56	0.73	0.90	100
	PTV	1	96.2	99.1	100	0.34	0.53	0.73	100
		2	99.5	99.9	100	0.29	0.47	0.64	100
		3	99.9	100	100	0.30	0.49	0.69	100
4D0	ITV	1	62.6	94.7	99.9	0.45	0.64	0.83	66.7
		2	80.9	97.6	100	0.53	0.69	0.87	82.7
		3	81.4	97.5	99.9	0.54	0.71	0.88	84
	PTV	1	77.2	96.6	100	0.29	0.42	0.62	75.3
		2	84.0	98.8	100	0.29	0.45	0.63	92.6
		3	93.3	99.0	100	0.29	0.48	0.68	93.8
4DN	ITV	1	42.8	85.1	99.3	0.14	0.50	0.81	21.9
		2	51.9	88.1	99.5	0.23	0.59	0.87	29.1
		3	48.2	87.3	99.7	0.26	0.62	0.87	30.1
	PTV	1	62.8	94.8	100	0.17	0.39	0.67	68.7
		2	65.8	96.8	100	0.21	0.44	0.70	81.2
		3	65.8	96.9	100	0.26	0.48	0.74	82.5

*Each margins case of the 3D0 calculations was done using the 3 foci described previously. In the case of 4D0 and 4DN values, the 9 possible focus/GW combinations presented previously were used*.

### 3.4 Tumor Motion and Size Dependence

**Figure 8:**
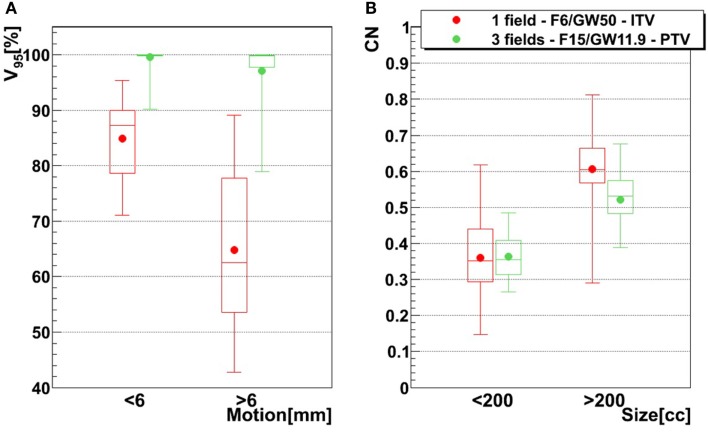
**Influence of motion magnitude on *V*_95_ tumor size on CN, using two different treatment configurations**. The red configuration represents simulations done using one field, a focus of 6 mm, a 50% GW, and ITV margins only, and the green configuration represents three fields, a focus of 15 mm, a 11.9% GW, and PTV margins (3 mm isotropic + 3 mm + 3% range margins). For the left graph **(A)**, bars representing tumors whose motion is lower than 6 mm are composed of 21 points (three patients, 21 weeks), while bars representing tumors whose motion is larger than 6 mm are composed of 49 points (six patients, 49 weeks). In the case of the right graph **(B)**, small tumor (<200 cc) bars are composed of 30 points (four patients, 30 weeks) and big tumor (>200 cc) bars are composed of 40 points (five patients, 40 weeks). See Table 1 for more details about patients.

The influence of the motion magnitude on *V*_95_ is displayed in Figure 8A. In case of the green combination (small focus, long GW, ITV, and one field), patients with a small motion (<6 mm) show an average *V*_95_ of 85%, as opposed to 65% for patients with a large motion. This difference of 20% is reduced to 3% if a large focus, a small GW, PTV margins, and 3 fields are used, which also yields mean *V*_95_ > 95% for both groups. Dependence of CN to the size is shown in Figure 8B. Patients with a smaller tumor (<200 cc) show mean CN of 25 and 16% lower than large tumors (>200 cc).

## 4 Discussion

In this study, a time series of 4DCTs of lung cancer patients was investigated for inter- and intrafractional effects of motion, anatomic changes, and setup errors. Most studies of particle therapy for moving targets focus on 4DCTs at a single time point, assuming nearly perfect treatment conditions. In this respect, the findings of this study offer highly important, previously unstudied information for a more clinically realistic scenario.

### 4.1 Beam Focus and Gating Window

Results show that the larger the focus and the shorter the GW, the better *V*_95_, meaning that intrafractional motion mitigation is more effective using a large focus and a short GW, as illustrated in Figure 4. Only the volume of the target (see Table 1 and Figure 8) seems to have a direct impact on CN: CN values yielded for simulations done with larger targets (patients 1, 2, 4, 5, and 6) are higher than values obtained for simulations done with smaller ones (patients 3, 7, 8, and 9); that can be observed in Figure 8 (and especially for the presented cases with ITV margins only). Thus, GW and focus do not show a significant influence on CN, which does not have a particular behavior regarding those parameters and is more patient specific: only patient 9 showed large weekly CN variability (range = 0.3) over the different GW/focus configurations compared to all other patients with a range < 0.1 [more details in Brevet (34)]. Thus, although *V*_95_ increases using a large focus and a short GW, the total volume to which 95% of the planned dose is delivered increases as well, i.e., OARs in the vicinity of the tumor are irradiated. In both studies by Steidl (18) and Richter (19), a larger focus permits to obtain better results in terms of target coverage, which is in agreement with what has been observed here. However, while a decreasing CN is obtained with increasing focus size in the study by Steidl (18), this behavior is not present in the study by Richter (19) and here. This can be explained by the fact that Steidl (18) used a different CN, which integrates the dose values obtained in all the voxels of the CTV and thus ignores the high interplay dependency of *V*_95_, the latter being itself the main component of the here used CN. Richter (19) shows that CN is decreasing with larger foci only for static cases (a behavior which can be also observed for static cases in Figure 3), while it is more patient specific for cases with motion and tends to converge on values obtained with static cases. The same behavior was observed in the study by Brevet (34): weekly results of each patient show that CN is patient and week specific and that no discernable trend for focus or GW can be found. As a global result, CN is slightly higher for larger foci, but when studied separately for each patient and each week, CN values do not show a systematic behavior. It can also be noticed that the focus size has a much more significant influence on the results compared to gating window. This is due to the size of the largest focus (15 mm), which is larger than the tumor motion for patients 1–8 or similar to the tumor motion for patient 9 (see Table 1). Hence, it is much easier to cover the moving tumor using this large focus. As using a small GW can increase treatment time considerably, this setting should be adjusted patient specifically.

Interfractional changes tend to dominate intrafractional ones, which can be reliably mitigated with gating and a large focus. The case shown in Figure 4 illustrates this (cf. Figures 4B,E), but also the dominant cause for interfractional dose errors: considerable change in range to the target. Though this depends on the chosen entry channel, Figures 4C,F show an extreme overshoot compared to the planned treatment dose. This is an extreme case, though, with considerable dose to OARs. On average, the impact is less severe, as can be seen by CN being restored nearly to the planned static value for multiple fields and large margins. An analysis of individual OARs and dose constraints would be necessary for more specific conclusions.

### 4.2 Margins

For intrafractional motion, Knopf et al. (25) and Albertini et al. (35), using different sorts of margins, confirmed that margins permit indeed to compensate efficiently for tumor motion. Here, additional margins to a range ITV were studied to recover misdosage from the interfractional changes. Figure 5 shows improving results when the irradiated volume is extended. Sorted by increasing order, isotropic, range, and combined isotropic/range margins yield better target coverage, for both 4D0 and 4DN simulations: *V*_95_ and CN are sensitive to additional ITV-PTV margins. *V*_95_ improves indeed significantly in terms of distribution range and mean value. And even though the effects of interfractional changes can still be observed for 4DN simulations (low minimal *V*_95_ value), using a combination of additional isotropic and range margins permits to increase the fraction of successful fractions from 21 to nearly 70%. It means that combining those two margins to extend the irradiated region improves coverage of the possible anatomic changes from fraction to fraction. CN, however, reduces due to some additional dose delivered in the vicinity of the target volume. This can be observed in Figures 4D–O: *V*_95_ is improved but the irradiated volume outside the tumor clearly increases gradually as more additional margins are used.

Figures 4D–F,M–O show how margins can allow dose recovery in the tumor for a patient with severe intra- and interfractional motion. In this case, the combination of range and isotropic margins permits to reach a mean *V*_95_ value 20% higher compared to the use of ITV margins only. Thus, the conclusions of Knopf et al. (25) and Albertini et al. (35), stating that intrafractional motion can be mitigated by the use of margins, can be extended by the fact that margins also allow to compensate efficiently for dose delivery deterioration caused by interfractional changes. However, it is also clearly visible that OARs, such as the spinal cord and the ipsilateral lung, are irradiated with a higher dose.

### 4.3 Number of Fields

To dilute the dose to OARs, multiple fields are typically employed and have also been shown to help mitigate intrafractional motion Knopf et al. (21) through an enhanced rescanning effect. Combined with ITV margins only, multiple fields significantly improve *V*_95_ and CN. Using two or three field did not result in an improvement in target coverage. This can be explained by the lack of an automatic optimization method for field directions. Using a generic, geometric approach to choose field directions, it became more likely with three fields to pass through tissue heavily affected by interfractional changes. Thus, a field affected, e.g., by a range shift would deteriorate target coverage instead of further improving it. This effect was minor and not significant, though. On the other hand, conformity could be further improved by distributing dose to more entry channels, which decreases *V*_95_ outside of the target and thus improves CN. This shows that choosing field directions carefully to avoid regions that are likely affected by interfractional changes would result both in good target coverage and good CN. Added PTV margins as expected improve the results further. *V*_95_ average values for 4DN simulations tend to converge to the values obtained for 4D0 simulations, showing that interfractional changes are almost completely mitigated. Outliers remain with an inadequate target coverage, but more than 90% of successful fractions become possible. CN is however drastically reduced due to the extended irradiated volume, which is now partly composed of normal tissue from the surrounding OARs. With the remaining difference of V95 between 4D0 and 4DN of 1.2% (range 0–21%), interfractional changes appear to be sufficiently compensated. Dose distributions in Figures 7A–I illustrate the great advantage of using three fields combined with additional margins. It allows obtaining a conformal dose distribution, with a target which is completely and homogeneously covered, and reduced high-dose regions outside the tumor. Again, field directions can be chosen differently to avoid or decrease further the irradiated volume of lung visible in Figures 7G–I.

This study has some limitations. The focus was set on identifying relevant planning parameters. To identify these, most plans would not be clinically valid or deliverable, but are helpful in showing the effect of isolated technical parameters. A further issue is the (unavoidable) use of deformable image registration to both propagate contours across the different CTs and phases and also to accumulate dose in the reference phase of each CT. Careful quality insurance was performed, using checker-board and false-color images as well as inspection of the resulting vector fields, with resulting errors to be expected in the order of 2 mm (36). To mimic patient setup, CTs were rigidly registered against each other, which might be more accurate than positioning with orthogonal X-rays, so that additional margins would be required. Finally, though serial 4DCT data was available, each 4DCT represented only a single breathing cycle. Variable breathing cycles could be studied using synthesized MR/CT data (37).

## 5 Conclusion

The aim of this study was to identify optimized treatment planning parameters in order to compensate for dose delivery deterioration caused by intrafractional tumor motion and interfractional variability. It was found that the use of a large focus (15 mm, FWHM), a short gating window (11.9% of the motion amplitude), ITV-PTV margins (3 mm isotropic + 3% + 3 mm range margins), and 3 fields yielded the best results in terms of target dose coverage. Less than 6% of fractions remained below 95%. In conclusion, in this first study combining state-of-the-art 4D dose calculation with serial 4DCT data, a combination of these parameters together with careful choice of field directions permits safe fractionated target dose coverage for NSCLC patients treated with ^12^C ions.

## Author Contributions

RB performed the simulations, evaluated the data, and wrote the manuscript. DR contributed to the study design, advised on 4D-dose calculation, and revised the manuscript. CG contributed to the study design and data evaluation and revised the manuscript. CB designed the study and revised the manuscript. MD supervised the study and extensively revised the manuscript.

## Conflict of Interest Statement

The authors declare that the research was conducted in the absence of any commercial or financial relationships that could be construed as a potential conflict of interest.
